# Impact of an 8-Month Trial Using Height-Adjustable Desks on Children’s Classroom Sitting Patterns and Markers of Cardio-Metabolic and Musculoskeletal Health

**DOI:** 10.3390/ijerph13121227

**Published:** 2016-12-10

**Authors:** Ana María Contardo Ayala, Jo Salmon, Anna Timperio, Bronwyn Sudholz, Nicola D. Ridgers, Parneet Sethi, David W. Dunstan

**Affiliations:** 1 Institute for Physical Activity and Nutrition (IPAN), School of Exercise and Nutrition Sciences, Deakin University, Burwood, VIC 3125, Australia; jo.salmon@deakin.edu.au (J.S.); anna.timperio@deakin.edu.au (A.T.); b.sudholz@deakin.edu.au (B.S.); nicky.ridgers@deakin.edu.au (N.D.R.); David.Dunstan@bakeridi.edu.au (D.W.D.); 2 Physical Activity Laboratory, Baker IDI Heart and Diabetes Institute, Melbourne, VIC 3004, Australia; Parneet.Sethi@bakeridi.edu.au; 3 Mary MacKillop Institute for Health Research, Australian Catholic University, Melbourne, VIC 3000, Australia

**Keywords:** sitting time, height-adjustable desks, school-age children, classroom-based intervention, musculoskeletal health, anthropometric measures, blood pressure

## Abstract

During school hours, children can sit for prolonged and unbroken periods of time. This study investigated the impact of an 8-month classroom-based intervention focusing on reducing and breaking-up sitting time on children’s cardio-metabolic risk factors (i.e., body mass index, waist circumference, blood pressure) and perceptions of musculoskeletal discomfort. Two Year-6 classes (24 students per class) in one primary school were assigned to either an intervention or control classroom. The intervention classroom was equipped with height-adjustable desks and the teacher was instructed in the delivery of pedagogical strategies to reduce and break-up sitting in class. The control classroom followed standard practice using traditional furniture. At baseline, and after 8-months, time spent sitting, standing, stepping, and sitting-bouts (occasions of continuous sitting) as well as the frequency of sit-to-stand transitions were obtained from activPAL inclinometers and the time spent in light-intensity physical activity was obtained from ActiGraph accelerometers. Demographics and musculoskeletal characteristics were obtained from a self-report survey. Hierarchical linear mixed models found that during class-time, children’s overall time spent sitting in long bouts (>10 min) were lower and the number of sit-to-stand transitions were higher in the intervention group compared to the control group, while no changes were observed for musculoskeletal pain/discomfort. No significant intervention effects were found for the anthropometrics measures and blood pressure. Height-adjustable desks and pedagogical strategies to reduce/break-up sitting can positively modify classroom sitting patterns in children. Longer interventions, larger and varied sample size may be needed to show health impacts; however, these desks did not increase musculoskeletal pain/discomfort.

## 1. Introduction

Accumulating evidence suggests that prolonged time spent in sedentary behaviours (i.e., sitting/reclining pursuits that involve an energy expenditure ≤1.5 metabolic equivalent units of rest (METs) during waking hours [1]), may be a distinct risk factor for cardiovascular diseases in adulthood, even after accounting for leisure-time moderate-to-vigorous intensity physical activity [2,3,4]. This is also true for studies with adults that objectively measured sedentary time [5,6,7,8]. There is, however, limited evidence of such associations in younger populations [9,10,11]. Most evidence is observational (i.e., cross-sectional or longitudinal) and few have found adverse associations between total sedentary time and adiposity, cardio-metabolic health risk markers or musculoskeletal outcomes, among children and adolescents [3,11,12]. 

Emerging research also indicates that the manner in which sedentary time is accumulated (e.g., prolonged bouts of sitting >15 min) has deleterious health consequences in adults [7,13,14]. While this association has been largely unexplored in children, one cross-sectional study reported that the accumulation of 5–10 min sitting bouts (i.e., defined as occasions of continuous sitting) was negatively associated with inflammatory markers (i.e., C-reactive protein) [15]. Thus, it is possible that the manner in which sedentary time is accumulated, rather than the total volume of sedentary time, may better explain associations between sedentary time and health outcomes in children and adolescents [16].

Recent experimental evidence indicates that breaking-up prolonged sitting with light-intensity physical activity (LPA) breaks (e.g., standing and stepping) can be beneficial for adults’ cardio-metabolic risk markers [14,17,18]. While these findings suggest that frequently interrupting sitting with LPA has the potential to minimise deleterious health effects associated with prolonged sitting time [19], only a few cross-sectional studies have examined this effect among younger populations [19,20,21,22]. Although these studies support the notion that interrupting long sedentary bouts may contribute to improved cardio-metabolic health in children [19,23], it is apparent that further experimental evidence is needed to better elucidate such relationships. 

During weekdays, children spend large amounts of their waking hours at school, where on average 50%–70% of this time is spent sitting [24,25,26]. In addition, during school hours, children have fewer breaks in sedentary time when compared with non-school time (e.g., 8.9 vs. 10.2 breaks per hour, respectively) [26,27]. However, whilst the length of classroom sitting-bouts remains unclear (e.g., from less than 5 min [15] to longer than 30 min [26]), and given the associations observed between bouts as short as 5–10 min and deleterious health outcomes [15], there is a need to identify strategies to both reduce total volume of sitting and the accumulation of prolonged sitting bouts during school hours.

Introducing height-adjustable desks into classroom settings has been suggested as a potential environmental strategy for reducing children’s sitting time and increasing their standing time during school hours [24,25,28,29,30]. This approach may also have a favourable impact on some health indicators such as energy expenditure [31,32], body mass index (BMI) [33] and musculoskeletal health [25,34]. However, a general consensus of the benefits of height-adjustable desks on health has not been reached [35]. Most studies have: typically involved short-term follow-ups (e.g., 18 weeks on average, with the exception of one study undertaken over 1.5 years [34]); had limited objective measurement of the entire physical activity spectrum (important for identifying what impact reducing sitting has on other intensities of physical activity); or just assessed adiposity-related outcomes (e.g., energy expenditure and BMI) or indices of musculoskeletal discomfort [36,37]. The potential impact of longer-term classroom sitting patterns (i.e., sitting bouts) on cardio-metabolic health (e.g., blood pressure (BP) and waist circumference (WC)) has not yet been studied in primary school students. 

Furthermore, previous studies using height-adjustable desks in classrooms have not included professional development for teachers to encourage and support students to use the desks, which may have reduced intervention effects [36,37]. Teacher-guided pedagogical approaches encouraging students to use the desks may enhance the effectiveness of such interventions on reducing and breaking-up sitting time. Previous research has examined the impact of teacher professional development sessions with pedagogical approaches during lessons in the absence of height-adjustable desks and observed a 13 min per day reduction in classroom sitting time [38,39]. However, the combination of pedagogical approaches and height-adjustable desks has not been investigated.

The aim of the current study was to examine the impact of an 8-month intervention incorporating height-adjustable desks and pedagogical strategies on overall volume and pattern of sitting time in the classroom, cardio-metabolic risk factors (BMI, WC, and BP) and perceptions of musculoskeletal discomfort among primary school children. 

## 2. Methods

The Make A Stand Kids (MASK) Study was a pilot non-randomised trial conducted in one government primary school in Melbourne. The intervention was conducted over an 8-month period from the beginning of Term One (March) to the beginning of Term Four (October) in 2014. The study protocol was approved by the Deakin University Research Ethics Committee (Project ID HEAG-H 93_2013) and The Department of Education and Early Childhood Development (Project ID 2013_002054). After the school principal provided written consent, two Year 6 classes (11–12 years old) at the school were selected and students in the classes were provided with an information pack for their parents/carers/guardians that included a plain language statement and a parental consent form for their child to participate in the evaluation component of the intervention. Parental consent to participate in the study was received from 48 students. Following this, one Year 6 class (24 students) was assigned as an intervention (IV) classroom and the other Year 6 class (24 students) as a control (CC) classroom. 

Each participant in the intervention class was provided with a manually adjustable height-adjustable workstation (LearnFit Ergotron Pty Ltd., Sydney, Australia) that allowed them to complete classwork in either a seated or standing position. Original classroom chairs were replaced with stools (Furnwear Bodyfurn Lab stool, Melbourne, Australia) high enough to allow a comfortable seated position for all participants when the desk was lowered. Prior to commencement of the intervention, a professional development session was delivered to the teacher in the intervention classroom, adapted largely from the Transform-Us! program [38]. This session covered: pedagogical approaches to reducing and breaking up sitting in the classroom which aimed to progressively increase the number of standing lessons and breaks in sitting time; how to adapt the delivery of their usual curriculum; and the safe and appropriate use of the desks. The pedagogical strategies recommended that extended classroom teaching blocks (of at least 60 min) be interrupted every 30 min with a two-minute guided light-intensity active break from sitting (e.g., bean-bag throwing between students while spelling/counting in a standing position). The teacher also delivered 9 key messages in the curriculum about the importance of reducing sitting and increasing physical activity. In addition, the teacher was asked to deliver at least one 30-min standing lesson each day [38]. The control class followed standard pedagogical practice and retained traditional classroom furniture. 

### 2.1. Measurements

All measurements occurred at two time points. Baseline assessments were conducted before the desks were placed in the classroom and repeated after 8 months (follow-up), during which the desks remained on the classroom. The measurements were taken by trained research staff, however, the same person did not conduct both the baseline and follow-up assessments. 

Children wore an activPAL inclinometer (PAL Technologies Ltd., Glasgow, UK) at the mid-point on the front of their right thigh during waking hours for eight consecutive days. This device (sampling at 10 Hz) detects limb position, and is a valid and reliable device for measuring sitting, standing and stepping time in primary school children [40,41]. In addition, participants wore an ActiGraph GT3X accelerometer (ActiGraph LLC, Pensacola, FL, USA) on a belt on their right hip for eight consecutive days. This device measures the acceleration and deceleration of human movement. Acceleration data can be used to identify the intensity of a given movement as well as its underlying pattern (i.e., frequency, duration) over predetermined epochs [41,42]. This device has demonstrated acceptable validity and reliability in children [43,44].

Systolic and diastolic BP measurements were taken three times on the participant’s right arm using an OMRON HEM-907 automatic digital blood pressure machine with a paediatric cuff. The first measure was performed after two minutes of seated rest with the remaining two measurements taken at 1-min intervals. The three BP measurements were averaged for a final value.

Children’s height was measured to the nearest 0.1 cm using portable stadiometers, and their body mass was measured to the nearest 0.1 kg using portable calibrated electronic scales. Waist circumference was measured using a flexible steel tape at the narrowest point between the bottom rib and the iliac crest, in the midaxillary plane. Two measurements were taken for height and waist circumference, respectively, for which the average of each was used; where a discrepancy of over 1 cm was apparent a third measurement was taken.

A brief questionnaire was administered in the classroom by research staff and supervised by the classroom teachers at baseline and at follow-up. Children were informed this was not a test and instructed to answer truthfully. The survey asked for socio-demographic characteristics (e.g., age, gender, cultural identity) and to assess location of any musculoskeletal pain/discomfort, based on the modified Nordic Musculoskeletal Questionnaire, which has been used in children previously [25,45,46]. Children were asked to report (yes/no) if they have at any time during last month had any trouble (ache/pain) in: shoulders, elbows, wrist/hands, hips/thigh, knees, ankles, neck, upper and low back. 

### 2.2. Data Management

All activPAL and ActiGraph data were downloaded using manufacturer proprietary software (activPAL Professional v7.2.29 and ActiLife v6.11.8, respectively) in 15-s epochs and processed using a customised Microsoft Excel macro. Non-wear time was defined as 20 min of consecutive zero counts, for both devices [47]. In the activPAL this was determined from the accelerometer rather than postural data. Participants were instructed to wear both monitors during all waking hours except during water-based activities. Children were included in the analysis if they had: (a) worn the activPAL and ActiGraph for at least three week valid days, defined as ≥8 h of wear time (weekend days were excluded in this analysis); and (b) if they had worn the monitors for at least 50% of each school period. For both devices, three time periods were extracted based on school bell times: (a) classroom time (i.e., time when participants were in classroom lessons, 300 min/day); (b) school time (i.e., classroom time and recess and lunch breaks, 390 min/day); and (c) waking hours (i.e., 960 min/day).

The average minutes that children spent sitting, standing, stepping, and in sitting bouts (>5, >10, >20 min duration) as well as the frequency of sit-to-stand transitions were obtained from the activPAL. No interruptions in the definition of sitting bouts were permitted in the data. Average minutes spent in LPA were obtained from the ActiGraph, using the Freedson age-adjusted accelerometer cut-off points [48,49]. Prior to the analysis, all activPAL and ActiGraph-derived outcome variables were standardised according to total wear time during the period of interest as follow: ((duration of X within period/wear time within period) multiplied by length of period), where X is the intensity/activity (e.g., sitting, standing, LPA).

BMI (kg/m^2^) and WC z-scores were calculated from raw anthropometric data using Stata functions (based on Cole (1990) method) [50]. Children’s BMI was categorised according to the International Obesity TaskForce definition of healthy weight or overweight/obese [51].

Questions related to the perception of musculoskeletal pain/discomfort were categorised into three body sites: (1) upper limbs (shoulders, elbows and wrist/hands); (2) lower limbs (hips/thigh, knees and ankles); and (3) back/spine (neck, upper and low back). All the answers were coded as 1 (yes) or 0 (no).

### 2.3. Statistical Analyses

Analyses were conducted using Stata 13.0 (StataCorp LP., College Station, TX, USA). Statistical significance was set at *p* < 0.05. Independent *t*-tests and Pearson chi-square tests were used to assess between group comparisons at baseline. Hierarchical linear mixed models were used (where unit of the analysis (students) were nested within clusters (classrooms)) to examine intervention effects on time spent sitting, standing, stepping, sitting bouts, LPA, and on sit-to-stand transitions frequency, as well as on BMI, WC and BP. Multilevel mixed-effects logistic regression models were used to examine intervention effects on the presence of pain/discomfort in the upper limbs, back/spine and lower limbs. All models were adjusted for baseline values to avoid regression to mean [52]. An intervention effect refers to the within-group change from baseline in the intervention group minus the within-group change from baseline in the control group. 

## 3. Results

### 3.1. Descriptive Characteristics

Valid activPAL data were obtained from 95% of the participants who wore the monitors in both groups at baseline, and 95% in the CC and 90% in the IV at follow-up. Additionally, valid ActiGraph data was obtained from 100% of the participants who wore the monitors in the CC and 95% in the IV at baseline, and 33% and 83% at follow-up, respectively. Anthropometric measures were obtained from 100% of participants at baseline and from 90% in the CC and 95% in the IV at follow-up. The musculoskeletal pain/discomfort questionnaire was answered by 95% in the CC and 100% in the IV at baseline and from all the participants at follow-up. 

Overall, there were no significant between-group differences at baseline for any demographics characteristics of any of the outcome measures (Table 1). At baseline, on average, pooled data showed that participants spent 62% of their waking hours sitting and 69% of their classroom time sitting, and 33% of waking hours and 34% of classroom time in LPA. 

### 3.2. Impact of the Intervention on Sitting and Activity Measures

The intervention effect and changes from baseline to follow-up in sitting and activity measures for both groups during classroom time, school time and waking-hours periods are shown in Table 2. A descriptive table, indicating the averages of time spent in all outcomes variables, is presented in Appendix (Table A1).

#### 3.2.1. During Classroom Time 

Compared to the CC group, the IV group spent significantly less time sitting in bouts of at least 10 min during classroom time, while there was a tendency (*p* = 0.06) for a greater frequency of sit-to-stand transitions in the IV relative to the CC. No significant intervention effects were found for the time spent sitting, standing, stepping or in LPA during classroom time (Table 2). 

#### 3.2.2. During School Time

The IV group spent significantly less time sitting in bouts of at least 10 min during school time and exhibited a significant positive mean difference in the frequency of sit-to-stand transitions relative to the CC group. Reductions in time spent in sitting bouts of >5 min and >20 min during school time were greater in the IV relative to the CC group, although these differences only approached statistical significance (Table 2). 

#### 3.2.3. Waking Hours

There were no intervention effects across the whole day (waking hours) for any of the outcome variables (Table 2).

### 3.3. Impact of the Intervention on Health Outcomes

#### 3.3.1. Anthropometric Measures and Blood Pressure 

Intervention effects and changes from baseline in anthropometric and blood pressure measurements are shown in Table 3. No significant intervention effects were found for BMI, WC or blood pressure, after the 8-months intervention period. 

#### 3.3.2. Perceived Musculoskeletal Pain/Discomfort

No significant intervention effects were found in relation to musculoskeletal pain/discomfort, after the 8-month intervention period (Table 4).

## 4. Discussion

To our knowledge this is the first study to have assessed the impact of an environmental intervention combining height-adjustable desks with pedagogical strategies to break up sitting during classroom lessons across a school year on objectively-measured sedentary/sitting time and LPA, classroom sitting accumulation patterns, children’s cardio-metabolic markers (BMI, WC and BP) and musculoskeletal health [36]. Overall, the intervention had a small positive influence on the manner in which the participants accumulated their sitting time during school hours, including less time spent sitting in long bouts (e.g., >10 min) and more sit-to-stand transitions compared to the control group. Importantly, the introduction of the desk did not increase musculoskeletal pain/discomfort.

A novel finding of this study was the significant impact of the desks on children’s sitting patterns in the classroom (i.e., less time spent in long sitting bouts and higher frequency of sit-to-stand transitions compared to the controls) and a positive trend towards higher standing and stepping (i.e., LPA). This is despite no changes in total classroom sitting volume being observed. Just as it is possible to determine health benefits from breaking up sitting independent of the volume of sitting [13], some intervention studies using objective postural devices have also shown it is possible to change the pattern of sitting without seeing corresponding changes in the volume of sitting [53]. This is possibly because of the small changes in sit-to-stand transitions, but could also be a reflection of how long children stood after each transition. Findings from cross-sectional studies in children and adolescents have suggested that prolonged sedentary bouts (i.e., lasting for more than 5 min) are detrimentally related to cardio-metabolic risk markers, with higher C-reactive protein [15], lower HDL cholesterol [23], higher BMI z-score [54] and higher BMI and WC in boys [55]. While our findings did not show any significant effects on cardio-metabolic markers from the intervention in this small sample of children over an 8-month period (which included 6 weeks of school holidays where children were not exposed to the desks), they do provide initial evidence of potentially beneficial changes in sitting patterns that may positively influence longer-term metabolic health among children. For example, the greater frequency of sit-to-stand transitions observed during classroom time in the intervention compared to the control group, may have favourable health benefits if such patterns could be sustained, since frequent interruptions of sedentary time have been positively associated with cardio-metabolic risk markers in children, adolescents as well in adults [14,19,23,56]. In addition, when morning recesses and lunchtime periods were considered in the analysis (i.e., the whole school day), the intervention effect on decreasing long sitting bouts and increasing the frequency of transitions was maintained. This indicates that the children in our study did not compensate for lower levels of sitting in class by sitting more during recess and lunch time.

In contrast to our findings, two previous studies involving primary school-aged children reported that the frequency of classroom sit-to-stand transitions decreased with access to height-adjustable desks [25,28]. However, both of those previous studies employed different approaches to the present study, notably they employed standing-biased desks (i.e., the desks were not adjustable and only allowed standing) and “active sitting” was encouraged when students were tired (e.g., Swiss balls, beanbags). Thus the observed decrease in the frequency of transitions in those studies may have resulted from participants choosing to engage in more prolonged standing time, leading to an overall reduction in postural transitions. Our findings are in agreement with previously observed research in secondary (high) school participants, which trialled the same height-adjustable desks as the current study and found more frequent short sitting bouts and fewer longer sitting bouts after a short intervention with height-adjustable desks [29].

In the analyses focusing on the whole day (including before, during and after school), none of the intervention effects remained significant. However, there was a tendency in the IV group to have greater reductions in time spent sitting in long bouts, compared with the CC group. This suggests that the changes due to the intervention might be too small to have a meaningful impact on overall daily sitting and light-intensity activities, or may be due to compensatory changes during non-school time (e.g., the effects of increasing a specific activity in school hours result in decreased activity in subsequent periods) [57]. However, further research is needed to establish whether compensation explains these results. 

No significant intervention effects were found for anthropometric (BMI and WC) or BP measures. Previously, a small study in primary school children (N = 8, mean age 11.3 ± 0.5 years) reported a similar, non-significant, small reduction in BMI after 8-months of standing desks use [33]. It is possible that intervention effects on anthropometric measures and blood pressure were non-significant due to the low intervention-related energy costs, inadequate length of exposure, and participants’ health profile. The use of height-adjustable desks has been associated with higher energy expenditure, compared to traditional classroom desks [32,58], suggesting a positive effect on adiposity markers (i.e., BMI and WC) and potential to influence childhood overweight and obesity. However, past research suggests that the energy cost of LPA (e.g., standing and stepping and used height-adjustable desks) may be too low [31,58,59] to expect short-term significant changes on total body mass and/or body fat distribution over this intervention period. Additionally, most of the participants (>80%) were categorised as having a healthy BMI and BP. Therefore, to better establish significant effects of height-adjustable desks on adiposity and cardio-metabolic markers in children, longer-term trials and more intensive breaks (e.g., moderate-intensity physical activity breaks) may be required, specifically amongst participants with more variable health profiles (e.g., higher BMI, WC and BP).

Previous studies have found that participants using height-adjustable desks reported more neck and back pain compared to control groups after the intervention [34], while others report less back and upper limb pain compared to baseline [25]. The current study found no significant intervention effects on the presence of pain/discomfort among participants, suggesting that using the height-adjustable desks for eight months did not increase musculoskeletal pain/discomfort after 8-months of intervention. Although previous studies also used self-reported data, it is important to note that various types of desks were employed (height appropriate standing workstation [25] and ergonomic furniture, including standing-biased desk [34]), and intervention durations also varied (from 2.1 to 18 months) which limits the comparability of results. Despite evidence suggesting that the prevalence of musculoskeletal problems in children can be reduced by increased standing time [34,60], inappropriate posture and long periods of standing can also lead to low back pain [61]. Therefore, it is reasonable to suggest that the introduction of height-adjustable desks should be accompanied by instructions on: (a) how to correctly use the desks (e.g., standing and sitting postures); (b) how to progressively increase standing time; and (c) education on strategies to avoid musculoskeletal discomfort such as foot shifting, feet rests, and stretching while standing. The latter strategies may even increase the activities associated with sitting breaks.

This is the first study to examine the impact of height-adjustable desks in combination with pedagogical strategies in primary school classrooms on reducing and breaking up sitting time. In children, the Transform-Us! intervention used pedagogical strategies (e.g., 2-min standing breaks from sitting and daily 30-min standing lessons) in conjunction with standing easels and observed a 13 min/day reduction in classroom sitting time among Australian 8–9 year olds after 6 months [39]. In contrast, the European intervention, UP4FUN, used curriculum-based education strategies only (without changes to pedagogy or the classroom environment) that focused on teaching children the importance of reducing sitting and increasing physical activity. There were no significant intervention effects on classroom sitting time among the 10–12 year olds in that study [62]. This suggests that modifications to the environment in addition to pedagogical strategies may require to achieve greater magnitude of change [35] and that curriculum-based strategies alone are unlikely to be sufficient. Future research should examine which strategies or combination of strategies are most effective at reducing and breaking-up sitting.

The major limitations of the present study are: (a) the small sample size; (b) the non-randomised study design; (c) loss of valid ActiGraph data and (d) lack of fidelity data (i.e., dose of exposure). (a) The small sample size reduced the statistical power and potential ability to identify differences in sitting and in the cardio-metabolic outcomes between groups over time; (b) The study was restricted to a comparison between two classrooms from a single school. As such, the magnitude of the intervention effect observed may be related to contamination of the intervention components (i.e., pedagogical strategies) from the intervention to the control classroom. For example, participants and staff shared common study and recreation areas during class time, which allowed for communication and experience exchanges between the participants and staff. Despite this, there were significant differences in sitting bouts of >10 min and in sit-to-stand transitions between the intervention and control classroom; (c) The results of this study may have been impacted by a disproportionate loss of ActiGraph data in the control group, whereby the control group provided considerably less valid data at follow-up, compared to the intervention group (i.e., 33% vs. 83% of the participants). However, more than 90% of participants provided valid activPAL data at both time points, which included stepping and standing time (i.e., LPA); (d) No observation data were collected regarding the fidelity of the intervention (e.g., how often the teacher implemented standing lessons), however, the information obtained from the activPAL inclinometers suggest that the desks were used by the children.

The present study included aspects not previously reported or considered in studies utilising similar interventions. These included: (a) a professional development session for the teacher of the children in the intervention classroom with pedagogical strategies to break-up sitting during lessons; (b) the impact of the intervention on the objectively measured sedentary/sitting time and LPA; (c) the analysis of sitting patterns accumulated during school hours (i.e., sitting bout lasted for 5, 10 and 20 min); and (d) the intervention impact on children’s WC and blood pressure. 

## 5. Conclusions

This study suggests that interventions that combine height-adjustable desks and pedagogical strategies to reduce/break-up sitting can positively modify classroom sitting patterns in children. The introduction of the desk did not increase perceived musculoskeletal pain/discomfort. To better establish the impact of height-adjustable desks on cardio-metabolic risk factors, further research should incorporate larger samples and longer-term cluster randomised controlled trials with multiple schools, varied age groups and examination of what factors prompted students to reduce and break-up their sitting during classroom lessons. 

## Figures and Tables

**Table 1:** Participant characteristics at baseline in control and intervention groups (mean ± standard deviation (SD), or percentages).

Descriptives	Control Mean (SD)	Intervention Mean (SD)	*p*-Value *
*N*	21	20	
Age (years)	11.7 (0.29)	11.5 (0.34)	0.10
Boys (%)	41.7	41.7	1.00
Cultural identity (%)			
Australian	78.3	90.5	0.37
BMI z-score (kg/m^2^)	0.09 (0.53)	0.33 (0.66)	0.18
BMI categories (%)			0.249
Normal weight	89	76.2	
Overweight	18	14.3	
Obese	0	9.5	
WC z-score (cm)	0.81 (0.89)	1.14 (0.88)	0.23
Systolic BP (mmHg)	108.8 (7.37)	110.9 (9.87)	0.46
Diastolic BP (mmHg)	61.7 (5.43)	60. 1 (6.30)	0.38
Sitting (min/day) (†)	609.5 (52.31)	588.5 (46.28)	0.18
LPA (min/day) (‡)	317.7 (46.74)	321.7 (34.9)	0.75
Presence of musculoskeletal pain (%) ^a^			
Upper limbs ^b^	29.1	45.8	0.21
Back/spine ^c^	33.3	25	0.51
Lower limbs ^d^	62.5	58.3	0.73

(†) Data obtained from activPAL inclinometers; (‡) Data obtained from ActiGraph accelerometers. ^a^ Percentage of participants that reported (i.e., yes) to musculoskeletal pain/discomfort in ^b^ Upper limbs (shoulders, elbows and wrist/hands); ^c^ lower limbs (hips/thigh, knees and ankles) and ^d^ back/spine (neck, upper and low back); * *p*-values are obtained using Pearson chi-square tests or two-sample T-tests as appropriate. BMI, body mass index; BP, blood pressure; min, minutes; mmHg, millimetre of mercury; *N*, number; LPA, light-intensity physical activity; WC, waist circumference.

**Table 2:** Intervention effects on children’s sitting time, sitting bouts of 5, 10 and 20 min duration, sit-to-stand transitions, standing, stepping and LPA for classroom time, school time and the whole day (waking hours).

Time Periods	Change between Baseline and Follow-Up						Intervention Effects (†)		
	Control			Intervention					
	Mean Change	95% CI	*p*	Mean Change	95% CI	*p*	Mean Difference	95% CI	*p*
Classroom time ^a,^*									
SIT (min)	−12.64	(−22.85, −2.44)	**0.015**	−21.34	(−31.55, −11.13)	**<0.001**	−8.69	(−23.13, 5.74)	0.238
SIT bouts >5 min	−13.57	(−24.43, −2.72)	**0.014**	−23.97	(−34.83, −13.12)	**<0.001**	−10.4	(−25.76, 4.96)	0.185
SIT bouts >10 min	−11.16	(−22.22, −0.11)	**0.048**	−28.83	(−40.56, −17.11)	**<0.001**	−17.67	(−33.78, −1.56)	**0.032**
SIT bouts >20 min	−10.64	(−21.76, 0.47)	0.060	−20.85	(−32.66, −9.04)	**0.001**	−10.21	(−26.72, 6.31)	0.226
STS transitions (freq)	−2.86	(−6.66, 0.94)	0.140	2.34	(−1.46, 6.15)	0.227	5.21	(−0.28, 10.7)	0.063
Standing (min)	11.56	(3.66, 19.46)	**0.004**	15.53	(7.63, 23.43)	**<0.001**	3.97	(−7.24, 15.19)	0.488
Stepping (min)	1.13	(−3.15, 5.4)	0.606	5.75	(1.47, 10.02)	**0.008**	4.62	(−1.46, 10.70)	0.136
LPA (min)	−9.3	(−20.28, 1.67)	0.096	−3.53	(−10.35, 3.29)	0.310	5.78	(−7.18, 18.73)	0.382
School time ^b,^*									
SIT (min)	−14.43	(−27.71, −1.16)	**0.033**	−23.87	(−37.15, −10.60)	**<0.001**	−9.44	(−28.25, 9.37)	0.325
SIT bouts >5 min	−13.9	(−28.9, 1.1)	0.069	−31.87	(−46.86, −16.87)	**<0.001**	−17.97	(−39.18, 3.25)	0.097
SIT bouts >10 min	−10.56	(−24.82, 3.71)	0.147	−38.3	(−53.43, −23.17)	**<0.001**	−27.75	(−48.54, −6.95)	**0.009**
SIT bouts >20 min	−10.84	(−24.31, 2.62)	0.115	−28.37	(−42.67, −14.07)	**<0.001**	−17.52	(−37.39, 2.34)	0.084
STS transitions (freq)	−7.9	(−12.16, −3.64)	**<0.001**	−0.64	(−4.90, 3.62)	0.768	7.26	(1.2, 13.32)	**0.019**
Standing (min)	20.97	(11.68, 30.25)	**<0.001**	24.63	(15.35, 33.91)	**<0.001**	3.66	(−9.49, 16.81)	0.586
Stepping (min)	−6.79	(−13.45, −0.12)	**0.046**	−0.55	(−7.21, 6.11)	0.871	6.24	(−3.26, 15.73)	0.198
LPA (min)	−9.24	(−22.57, 4.07)	0.174	−4.4	(−12.68, 3.89)	0.298	4.85	(−10.87, 20.57)	0.546
Waking hours ^c,^*									
SIT (min)	−42.98	(−74.73, −11.23)	**0.008**	−23.83	(−56.56, 8.92)	0.154	19.15	(−26.8, 65.1)	0.414
SIT bouts >5 min	−22.55	(−56.33, 11.23)	0.191	−41.15	(−75.97, −6.33)	**0.021**	−18.6	(−67.13, 29.92)	0.452
SIT bouts >10 min	−14.48	(−44.99, 16.02)	0.352	−51.98	(−85.41, −18.55)	**0.002**	−37.5	(−82.84, 7.83)	0.105
SIT bouts >20 min	−19.03	(−49.4, 11.33)	0.219	−31.62	(−65.12, 1.88)	0.064	−12.58	(−59.51, 34.35)	0.599
STS transitions (freq)	−12.22	(−12.22, −4.46)	**0.002**	−2.35	(−10.35, 5.65)	0.564	9.87	(−1.31, 21.05)	0.084
Standing (min)	37.25	(20.77, 53.73)	**<0.001**	26.75	(9.75, 43.74)	**0.002**	−10.5	(−34.25, 13.24)	0.386
Stepping (min)	5.6	(−12.41, 23.6)	0.542	−2.87	(−21.43, 15.69)	0.762	−8.47	(−34.42, 17.48)	0.522
LPA (min)	−15.28	(−39.75, 9.18)	0.221	−19.16	(−35.32, −3.00)	**0.020**	−3.88	(−33.24, 25.48)	0.796

(†) Intervention effect refers to the within group change from baseline in the intervention group minus the within group change from baseline in the control group based on hierarchical linear mixed models. All models were standardized according to total wear time during the period of interest, were adjusted for baseline values to avoid regression to mean and no-adjusted by sex (no differences by sex were detected). Significant differences (*p* < 0.05) are highlighted in **bold**. ^a^ Classroom time: 300 min per day; ^b^ School time: 390 min per day; ^c^ Waking hours: 960 min per day; * Data obtained from activPAL inclinometers except for LPA that was obtained from ActiGraph accelerometers. SIT, sitting; STS, sit-to-stand transitions; LPA, light-intensity physical activity; min, minutes; freq, frequency; 95% CI: 95% confident interval.

**Table 3:** Intervention effects on anthropometrics and blood pressure measurements.

Health Outcomes	Changes between Baseline and Follow-Up						Intervention Effects (†)		
	Control Group			Intervention Group					
	Mean Change	95% CI	*p*	Mean Change	95% CI	*p*	Mean Difference	95% CI	*p*
**Anthropometry**									
BMI z-score (kg/m^2^)	−0.23	(−0.44, −0.2)	**0.03**	−0.16	(−0.37, 0.06)	0.152	0.07	(−0.23, 0.38)	0.624
WC z-score (cm) ^a^	0.08	(−0.10, 0.29)	0.391	0.29	(0.10, 0.48)	**0.002**	0.21	(−0.05, 0.48)	0.118
**Blood pressure**									
Systolic BP (mmHg) ^b^	1.29	(−1.47, 4.06)	0.358	2.45	(−0.23, 5.15)	0.073	1.16	(−2.7, 5.02)	0.555
Diastolic BP (mmHg) ^c^	−2.1	(−4.55, 0.34)	0.091	−4.24	(−6.62, −1.86)	**<0.001**	−2.13	(−5.56, 1.29)	0.223

(†) Intervention effect refers to the within group change from baseline in the intervention group minus the within group change from baseline in the control group based on hierarchical linear mixed models. Significant differences (*p* < 0.05) are highlighted in **bold**. Technical error of measurements, for baseline and follow-up respectively are: ^a^ WC = 0.4 and 0.32 cm; ^b^ Systolic BP = 4.5 and 4.11 mmHg; ^c^ Diastolic BP = 4.02 and 4.49 mmHg. BMI, body mass index; BP, blood pressure; WC, waist circumference; mmHg, millimetre of mercury; 95% CI: 95% confident interval.

**Table 4:** The odds ratios (95% confidence internal (95% CI)) of reporting musculoskeletal pain/discomfort from the intervention.

Body Section	Odds Ratio *	95% CI	*p*
Upper limbs ^a^	0.79	(0.18, 3.47)	0.758
Back ^b^	1.48	(0.38, 5.72)	0.567
Lower limbs ^c^	1.24	(0.3, 5.1)	0.765

* Odd ratios obtained from multilevel mixed-effects logistic regression models, adjusted for baseline values to avoid regression to the mean. Self-report of aches/pain during last month in: ^a^ upper limbs (shoulders, elbows and wrist/hands); ^b^ lower limbs (hips/thigh, knees and ankles) and ^c^ back/spine (neck, upper and low back).

## Abbreviations

The following abbreviations are used in this manuscript:

IV	intervention group
CC	control group
BMI	Body mass index
BP	blood pressure
WC	waist circumference
SIT	sitting
STS	sit-to-stand
LPA	light intensity physical activity
95% CI	95% confident interval

## Appendix A

**Table A1:** Average time spent in sitting time, sitting bouts of 5, 10 and 20 min duration, sit-to-stand transitions, standing, stepping and LPA for classroom time, school time and the whole day (waking hours) for the control and intervention group at baseline and follow-up.

Time Periods	Control		Intervention	
	Baseline	Follow-Up	Baseline	Follow-Up
	Mean (SD)	Mean (SD)	Mean (SD)	Mean (SD)
Classroom time ^a,^*				
SIT (min)	207.29 (16.16)	191.24 (20.84)	204.99 (13.27)	181.80 (26.95)
SIT bouts >5 min	153.48 (17.99)	138.64 (20.41)	153.27 (18.10)	128.02 (28.11)
SIT bouts >10 min	122.03 ( 20.4)	107.71 (23.22)	123 (23.11)	95.9 (31.26)
SIT bouts >20 min	81.52 (24.57)	64.15 (24.35)	69.87 (29.6)	55.11 (30.87)
STS transitions (freq)	31.97 (6.8)	29.87 (6.33)	34.85 (7.32)	36.24 (10.22)
Standing (min)	49.63 (10.73)	64.14 (17.52)	49.93 (9.63)	66.49 (20.13)
Stepping (min)	43.19 (9.51)	44.71 (10.89)	45.19 (10.83)	51.83 (13.40)
LPA (min)	100.94 (22.5)	87.76 (21.1)	99.13 (17.2)	95.84 (19.77)
School time ^b,^*				
SIT (min)	236.03 (24.15)	219.25 (26.95)	232.54 (25.5)	206.21 (38.64)
SIT bouts >5 min	182.25 (29.71)	164.26 (33.24)	179.76 (30.05)	146.02 (37.16)
SIT bouts >10 min	143.14 (30.66)	126.93 (34.09)	142.9 (32.9)	106.68 (38.07)
SIT bouts >20 min	95.31 (30.14)	75.95 (30.5)	81.92 (34.31)	60.72 (36.4)
STS transitions (freq)	44.14 (7.14)	37.93 (6.25)	46.71 (8.63)	44.51 (12.14)
Standing (min)	72.11 (13.22)	94.85 (21)	71.89 (13.84)	97.18 (23.99)
Stepping (min)	82.02 (21.57)	76.03 (16.11)	85.72 (23.65)	86.76 (22.17)
LPA (min)	141.59 (24.86)	128.94 (24.82)	140.27 (20.1)	136.23 ( 24.75)
Waking hours ^c,^*				
SIT (min)	609.5 (52.31)	558.72 (52.12)	588.59 (46.28)	565.53 (90.67)
SIT bouts >5 min	485.39 (50.77)	455.47 (61.22)	476.91 (45.94)	436.85 (89.04)
SIT bouts >10 min	392.29 (52.22)	368.81 (65.05)	384.73 (52.15)	340.28 (84.51)
SIT bouts >20 min	262.14 (50.37)	234.54 (60.71)	225.42 (58.96)	209.47 (83.28)
STS transitions (freq)	101.66 (14.19)	90.1 (13.9)	103.72 (16.63)	101.59 (23.91)
Standing (min)	173.57 (28.26)	217.93 (35.58)	187.69 (33.06)	213.13 (53.20)
Stepping (min)	177.27 (41.61)	183.64 (32.79)	184.06 (45.26)	181.65 (55.81)
LPA (min)	317.74 (46.74)	301.76 (47.92)	321.72 (34.9)	299.71 (46.32)

All values were standardized according to total wear time during the period of interest. ^a^ Classroom time: 300 min per day; ^b^ School time: 390 min per day; ^c^ Waking hours: 960 min per day. * Data obtained from activPAL inclinometers except for LPA that was obtained from ActiGraph accelerometers. SIT, sitting; STS, sit-to-stand transitions; LPA, light-intensity physical activity; min, minutes; SD, standard deviation.
